# Should prophylactic thrombolysis be routine in clinical practice? Evidence from an autopsy case of septicemia

**DOI:** 10.1186/1472-6890-14-6

**Published:** 2014-01-30

**Authors:** Kunihiro Inai, Sakon Noriki, Hiromichi Iwasaki

**Affiliations:** 1 Division of Molecular Pathology, Department of Pathological Sciences, University of Fukui, 23-3 Matsuoka-Shimoaizuki, Eiheiji, Fukui 910-1193, Japan; 2 Division of Tumor Pathology, Department of Pathological Sciences, University of Fukui, 23-3 Matsuoka-Shimoaizuki, Eiheiji, Fukui 910-1193, Japan; 3 Autopsy Imaging Center, School of Medical Sciences, University of Fukui, 23-3 Matsuoka-Shimoaizuki, Eiheiji, Fukui 910-1193, Japan; 4 Division of Infection Control, University of Fukui Hospital, 23-3 Matsuoka-Shimoaizuki, Eiheiji, Fukui 910-1193, Japan

**Keywords:** Autopsy, Candida spp, Central venous catheter, Sepsis, Thrombus, Pathological staining

## Abstract

**Background:**

Central venous catheters provide easy access for intravenous infusion and nutrition, but they can bring about complications such as catheter-related infections. Infected central venous catheters often cause nosocomial bloodstream infections with high morbidity and mortality. However, most of the morphological data that have been published are derived from *in vitro* and *in vivo* studies and few reports of direct evidence obtained from patient-derived samples have been described. Here we present visual evidence of catheter-related candidemia. To our knowledge, this is the first reported conventional histopathological evidence of a *Candida*-infected intraluminal thrombus in a patient’s central venous catheter.

**Case presentation:**

A 62-year-old Japanese female with obstructive jaundice, gastrointestinal bleeding, and liver metastasis from pancreatic head cancer was given an implantable subcutaneous central venous port for nutrition and chemotherapy administration. High fever ensued on day 16 after the central venous port insertion and blood cultures revealed *Candida albicans.* Although the patient was given 300 mg/day of fosfluconazole according to the suggestion of the infection control team, she died from respiratory failure. Postmortem computed tomography revealed findings consistent with acute respiratory distress syndrome, suggesting that the patient’s course was complicated by catheter-related sepsis. Autopsy revealed a subcutaneous abscess around the port, from which *C. albicans* was cultured. However, no catheter-adherent thrombus, thrombosis of the great central veins, or endocardial vegetations were detected in the patient. Histological analysis revealed scattered abscesses in several organs including lungs and kidneys. Hyaline membrane formation and *Candida* colonies were found in the lungs. The central venous port tube, together with the part of the subclavian vein into which it had been inserted, was involved in an intraluminal fibrin thrombus containing neutrophils and macrophages, indicating that the thrombus existed while the patient was alive. Histopathological examination following use of the periodic acid-Schiff reagent and the Grocott stain revealed scattered *Candida* in the thrombus.

**Conclusions:**

Prophylactic thrombolysis should be encouraged to prevent central venous catheter-related candidiasis in clinical practice.

## Background

Although central venous catheters (CVCs) provide easy access for intravenous infusion and total parenteral nutrition, they can induce complications such as pneumothorax, hemorrhage, nerve injury, extravasation of infused material, venous thrombosis, cardiac arrhythmias, and catheter-related infections [1,2]. Infected CVCs often induce nosocomial bloodstream infections that are associated with high morbidity and mortality rates. The most common causative microorganism is coagulase-negative staphylococcus due to its capacity to form adherent biofilms [3]. Studies of biofilm-producing bacteria have mostly involved microbiological analyses. Therefore, visual data are limited to coagulase-negative staphylococci revealed by electron microscopy [4]. *Candida* is another critical pathogen that infects the bloodstream via the formation of biofilms on the surfaces of indwelling medical devices [5]. *Candida* can proliferate on catheter tips and on clots adhering to catheter tips [6]. There have been only a few previous reports of candidal septic thrombus and thrombophlebitis in the central veins [7-11], and they are rarely diagnosed while the patient is alive. Autopsies have revealed thrombus within cannulated central veins in up to half of all catheterized patients [12]. However, most of the reported morphological data regarding these lesions are derived from *in vitro* and *in vivo* studies. Hence, there is little reported evidence obtained from patient-derived samples. Here, we present visual evidence of catheter-related candidemia after staining for antigens present in the intraluminal catheter thrombus.

## Case presentation

A 62-year-old Japanese female was found to have obstructive jaundice secondary to pancreatic head cancer. A stent was placed endoscopically to relieve the biliary obstruction. Although chemotherapy was performed, the tumor metastasized to the liver. Subsequently, obstruction by the head of the pancreas occurred, which caused gastrointestinal bleeding. Therefore, an implantable subcutaneous central venous (CV) port was inserted for administration of nutrition and chemotherapy. Intravenous hyperalimentation was given by CV port over 24 h. Thus, lock therapy was not pursued in this patient. On day 13 after the CV port insertion, a low-grade fever developed. High fever and dyspnea ensued on day 16, and blood cultures revealed *Candida albicans* (*C. albicans*). White blood cells were 7 × 10^3^/μl at the onset of candidemia followed by the gradual increase up to 13.3 × 10^3^/μl before death, indicating that the patient was not neutropenic during the hospitalization. Her attending physicians believed the *Candida* infection occurred via the biliary stent. Therefore, antemortem echocardiography was not performed and the indwelling CV port was used throughout the hospitalization period. The patient was treated with 300 mg/day of fosfluconazole and 1 g/day of doripenem via the CV port. The use of fosfluconazole was per the suggestion of the infection control team; this drug is the first-line antifungal treatment of *C. albicans* in Japan. In addition, the isolated pathogen was the fosfluconazole-sensitive strain. In spite of these efforts, the patient died on day 26 from respiratory failure. Written, informed consent was obtained from a family member of the deceased to perform both postmortem computed tomography (CT) and autopsy. These analyses were approved by the ethics review board of the University of Fukui Hospital.

**Figure 1:**
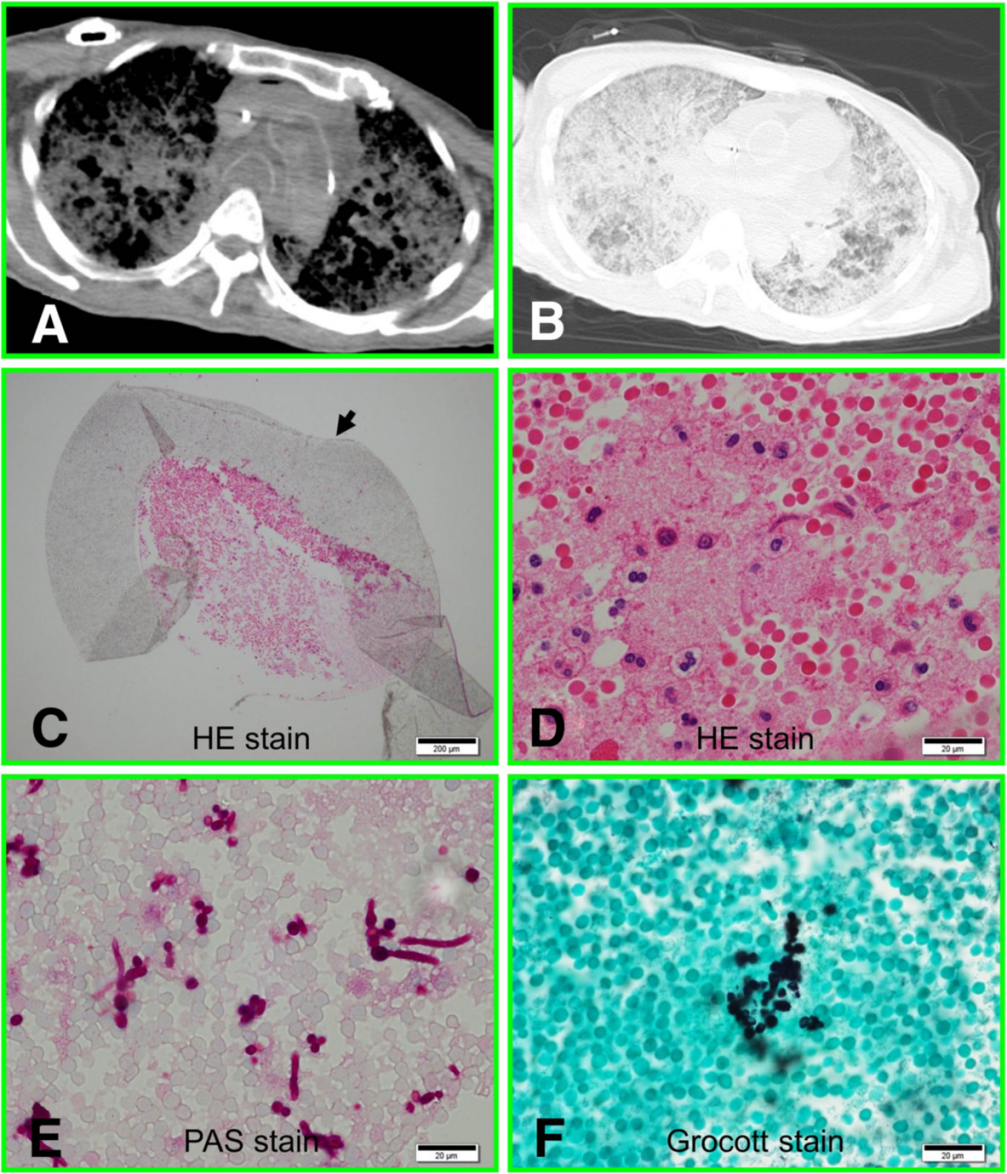
**Postmortem analysis. A)** Postmortem CT of mediastinal window. **B)** Postmortem CT of lung window. **C)** Fibrin thrombus in the CV port tube (arrow). **D)** High-magnification view of the thrombus containing neutrophils and macrophages. **E)***Candida* in the thrombus stained by the periodic acid-Schiff reagent. **F)** Detection of *Candida* by Grocott staining.

To analyze the pathogenesis in this patient, postmortem investigations were performed at the Autopsy Imaging Center of the University of Fukui. A postmortem CT scan was performed using an 8-slice multidetector CT used exclusively for the body. A full-body scan from the vertex to the feet was performed in the supine position to obtain volumetric CT data. The scanning conditions were: 120 kV, 250 mA, collimation 8 × 2.5, pitch 1.125, rotation time 0.8 s, slice thickness 5 mm, and increment 5 mm. Postmortem CT revealed a diffuse infiltration shadow widely distributed in the lung fields bilaterally. Two certified radiologists independently interpreted the images as consistent with acute respiratory distress syndrome (Figure 1A and B), suggesting that the patient’s course was complicated by catheter-related sepsis. Subsequently, a medical autopsy was performed by a certified pathologist and histological data were analyzed by two independent pathologists. No catheter-adherent thrombus, thrombosis of the great central veins, or endocardial vegetations were detected by gross section views. Histological analysis revealed scattered abscesses in several organs including lungs and kidneys. Infectious cholangitis was not detected. In addition, hyaline membrane formation and *Candida* colonies were found in the lungs. These histological findings confirmed the suspected ARDS due to *Candida* septicemia. A skin incision revealed a subcutaneous abscess around the CV port, from which *C. albicans* was cultured. The CV port tube, together with the part of the subclavian vein into which it had been inserted, were fixed with formalin, embedded in paraffin, sectioned and stained with hematoxylin-eosin. A cross-section of the catheter that had been inserted into the subclavian vein revealed a fibrin thrombus (Figure 1C) containing neutrophils and macrophages (Figure 1D), indicating that the thrombus had existed while the patient was alive. Staining with the periodic acid-Schiff reagent and the Grocott stain revealed scattered *Candida* with spores and elongated pseudomycelia in the thrombus (Figure 1E and F). The pathogen was detected from two distinct areas of the catheter tube. In contrast, by light microscopy, the outer wall of the catheter was clear, without fixations (arrow, Figure 1C).

## Discussion

Candidemia is a life-threatening nosocomial infection with high morbidity and mortality, especially in immunocompromised and critically ill patients [13], and indwelling CVCs have been identified as an independent risk factor for bloodstream infection [14]. We described a patient’s course which was complicated by *C. albicans* septicemia due to an implanted CV port, and death occurred following development of sepsis, including systemic inflammation and acute respiratory distress syndrome. The patient had no thrombosis in the great central veins, valvular vegetations, or cholangitis on autopsy, suggesting that the CV port became an entry site for the fungi and that the microbes were spread hematogenously.

Catheter-related thrombosis of the central veins is known to be a frequent but mostly subclinical complication of central venous lines [6]. *Candida* grows on the catheter tip or on the clot adherent to the catheter tip, especially in patients with catheter-related thrombosis, thrombophlebitis, and endocarditis [6-8,11]. Meanwhile, our patient exhibited the intraluminal catheter thrombus only and didn’t have any thrombosis in the central veins or endocarditis. The extent of intraluminal surface coverage by biofilm and the adherence of crystalized deposits are greater than for extraluminal surfaces [2,15]. Therefore, the thrombus formation may be initiated on the catheter tip or in the intraluminal space of CVCs.

Fluconazole is a standard therapeutic agent for candidemia and invasive candidiasis [16]. Particularly in Japan, it is used as the first-line therapy for drug-sensitive *C. albicans*. Thus, fluconazole was administered to the patient; in spite of this treatment, the patient died. As for therapy for candida thrombophlebitis, a rare but life-threatening complication of CVCs, a recent review recommends prolonged use of amphotericin B or echinocandins [7]. Hence, these drugs may be preferentially administered to patients with CVC-related candidemia, even those infected with the fluconazole-sensitive strains of *C. albicans*.

Although several experimental and clinical studies have involved providing prophylaxis against CVC-related infections [17-21], reports regarding histopathological analysis of catheter-related infections are lacking in the literature, except for those of a few surgically-resected thrombi. In general, histopathological data have high value as evidence because they are visible. However, there have been few such analyses of clinically-used CVCs, except for the detection of biofilm formation by electron microscopy. The reason is presumably that microbes such as bacteria are less likely to be detected by conventional histopathological techniques. However, there are only a few facilities that have ready access to electron microscopy on the premises. On the other hand, the size of fungi, including *Candida*, *Aspergillus*, and *Zygomycota,* is larger than that of bacteria. Therefore, we attempted to detect the intraluminal thrombus using standard pathological methods and were able to diagnose this rare case. Thus, in addition to using the roll-plate culture of CVCs [22], pathologists should analyze thrombi for fungi using conventional stains, particularly in cases with catheter-adherent thrombus or intraluminal thrombus.

Prophylactic methods are mainly divided into 3 different approaches: (a) systemic antifungal or antibiotic prophylaxis [20]; (b) protection from microbial colonization using antimicrobial-coated catheters[19], antibiotic locks such as vancomycin, macrolides [3] or taurolidine-citrate [18]; and (c) prevention of thrombosis using urokinase rinses[21] and flushing the catheter lumen with heparin solution [17]. In our case, the fibrin thrombosis growing *Candida* was clearly detected by medical autopsy. In addition, the thrombus had formed while the patient was alive. This fact indicates that the thrombus became a focus of the *Candida* infection. Thus, prophylactic thrombolysis should be further investigated for potential use in routine clinical practice as an important measure to prevent catheter-related *Candida* infection.

## Conclusions

To our knowledge, this is the first reported histopathological evidence of a *Candida* infection of an involved intraluminal thrombus in a patient’s indwelling CVC, as visualized by conventional histological staining. The visual evidence of catheter-related candidemia, obtained by staining the intraluminal catheter thrombus for antigens, indicates that prevention of thrombosis should play a central role in routine clinical practice as prophylaxis for catheter-related candidiasis.

## Consent

Written informed consent was obtained from a family member of the deceased patient for publication of this case report and any accompanying images. A copy of the written consent is available for review by the Editor of this journal.

## Abbreviations

Candida albicans: *C. albicans*; CT: Computed tomography; CVC: Central venous catheter.

## Competing interests

The authors declare that they have no competing interests.

## Authors’ contributions

KI contributed to the autopsy, autopsy data analysis, writing and review of the paper. SN contributed to autopsy data analysis and review of the paper. HI contributed to patient care and review of the paper. All authors read and approved the final manuscript.

## Pre-publication history

The pre-publication history for this paper can be accessed here:

http://www.biomedcentral.com/1472-6890/14/6/prepub
